# P2Y Receptor Modulation of ATP Release in the Urothelium

**DOI:** 10.1155/2014/830374

**Published:** 2014-04-14

**Authors:** Kylie J. Mansfield, Jessica R. Hughes

**Affiliations:** Graduate School of Medicine and Illawarra Health and Medical Research Institute, University of Wollongong, Wollongong, NSW 2522, Australia

## Abstract

The release of ATP from the urothelium in response to stretch during filling demonstrates the importance of the purinergic system for the physiological functioning of the bladder. This study examined the effect of P2 receptor agonists on ATP release from two urothelial cell lines (RT4 and UROtsa cells). Hypotonic Krebs was used as a stretch stimulus. Incubation of urothelial cells with high concentrations of the P2Y agonist ADP induced ATP release to a level that was 40-fold greater than hypotonic-stimulated ATP release (*P* < 0.0011, ADP EC50 1.8 µM). Similarly, an increase in ATP release was also observed with the P2Y agonist, UTP, up to a maximum of 70% of the hypotonic response (EC50 0.62 µM). Selective P2 receptor agonists, **αβ**-methylene-ATP, ATP-**γ**-S, and 2-methylthio-ADP had minimal effects on ATP release. ADP-stimulated ATP release was significantly inhibited by suramin (100 µM, *P* = 0.002). RT4 urothelial cells break down nucleotides (100 µM) including ATP, ADP, and UTP to liberate phosphate. Phosphate liberation was also demonstrated from endogenous nucleotides with approximately 10% of the released ATP broken down during the incubation. These studies demonstrate a role for P2Y receptor activation in stimulation of ATP release and emphasize the complexity of urothelial P2 receptor signalling.

## 1. Introduction

The first evidence for P2 receptor signalling from the urothelium was provided in 1997 with the work of Ferguson et al., who demonstrated in tissue strips that ATP is released from the urothelium in response to stretch [1]. Our understanding of the sensory role of the urothelium has since greatly increased, with stretch-induced ATP release from the urothelium also identified in cultured cells [2] and* in vivo* preparations [3].

ATP binds to two families of P2 receptors, P2X ligand-gated ion channels and G protein-coupled P2Y receptors. To date seven P2X receptors have been identified [4], with P2X_4_ and P2X_2_ receptors [5] expressed on the urothelium and P2X_3_ expressed on the urothelium [5, 6] as well as on suburothelial afferent nerves and myofibroblasts [7]. It is hypothesised that ATP released from the urothelium interacts with P2 receptors located on both afferent nerves (P2X_3_) [8] and myofibroblasts to signal bladder fullness. A role for ATP in bladder dysfunction has been postulated with increased ATP release associated with sensory disorders such as interstitial cystitis [9] and painful bladder syndrome [10]. Furthermore, ATP has been shown to play a role in bladder sensation, with ATP concentration in the intravesical fluid correlating with the first desire to void in patients with both overactive bladder [3] and painful bladder [11] but not in control patients.

P2X receptors are activated preferentially by ATP; thus urothelial derived ATP may have an autocrine action on urothelial P2X receptors. However the urothelium also expresses ectoATPase enzymes [12] and so is able to hydrolyse ATP (and other nucleotides) to breakdown products such as ADP, AMP, and adenosine. The perceived function of these enzymes is to limit the availability of ATP. Recently, the urothelium has been shown to release nucleotides other than ATP with twelve nucleotides quantified by HPLC in patient urine specimens [13]. The levels of ADP, UTP, UDP, and GTP were found to be more than 10 times higher than the level of ATP [13]. Other epithelial cells have also been shown to release nucleotides such as ATP, ADP, AMP, adenosine, UTP, and UDP, in response to hypotonic stimulus [14]. The role of these nucleotides in P2 receptor signalling in the bladder is yet to be determined. It is known that in addition to ATP, other nucleotides, in particular ADP and UTP, can also be substrates for P2Y receptors; however, their effects on urothelial P2Y receptors have not been explored. Eight P2Y receptors have been identified [15] with P2Y_1_ [5, 16], P2Y_2_ [5, 16, 17], P2Y_4_ [16, 17], and P2Y_11_ [5] expressed on urothelial cells. P2Y_6_ receptors are expressed on suburothelial myofibroblasts [18] but their expression on urothelial cells remains controversial [5, 19]. The function of these receptors on the urothelium is currently undetermined. In addition, adenosine formed from the breakdown of ATP binds to P1 receptors which are also expressed on the urothelium [20].

It is very clear that the complexity of urothelial P2 receptor signalling and the feedback of ATP breakdown and release have not been adequately explored to date. Therefore the aims of this study were to examine the effect of purine and pyrimidine nucleotides on urothelial cell ATP release. We hypothesise that purines and pyrimidines selective for P2Y receptors will modulate ATP release from the urothelium.

## 2. Materials and Methods

### 2.1. Cell Culture

Human urothelial RT4 cells were obtained from the ECACC. Cells were grown at 37°C with 5% CO_2_ in McCoy's 5A culture medium supplemented with 10% foetal bovine serum, 100 units/mL of penicillin, 100 *μ*g/mL of streptomycin, and 0.25 *μ*g/mL of fungizone. When confluent, RT4 cells were passaged with 0.05% Trypsin-EDTA for 5 minutes and then plated onto T75 flasks for continuous passage or onto 24 well plates for use in experiments when confluent (approximately 3 to 5 days after passage).

Human urothelial UTOtsa cells were a gift from Dr. Scott Garrett from the University of North Dakota. Cells were grown at 37°C with 5% CO_2_ in Dulbecco's modified eagle's medium supplemented with 5% foetal bovine serum, 100 units/mL of penicillin, 100 *μ*g/mL of streptomycin, 0.25 *μ*g/mL of fungizone, and 1% glucose. UROtsa cells were passaged in the manner described above for RT4 cells.

### 2.2. ATP Release Studies

ATP release was determined as we have described previously [2]. Urothelial cells were washed (three times) with carbogenated Krebs-Henseleit solution (containing mM: NaCl 118, KCl 4.7, NaHCO_3_ 25, KH_2_PO_4_ 1.2, MgSO_4_ 1.2, CaCl_2_ 2.5, and D-glucose 11.7). The basal level of ATP release was then determined by a 10-minute incubation in 500 *μ*L Krebs-Henseleit solution. These media were collected and followed with the experimental treatments. Cells were exposed to normal Krebs-Henseleit (control) or the indicated concentration of nucleotide. Cells were exposed to hypotonic Krebs-Henseleit (1 : 2 dilution of Krebs-Henseleit in distilled water), a stimulus commonly used to mimic stretch in cultured cells [2]. Cells were treated for 10 min before the supernatant (200 *μ*L) was collected and used for ATP determinations.

ATP concentration in the supernatant was measured using the bioluminescence assay according to the manufacturer instructions. Equal volumes of the cellular supernatant or ATP standard solutions (10^−6^ to 10^-10 ^M) were mixed with the bioluminescence assay mix and the luminescence generated was measured immediately using a plate reader (BMG labtech Polarstar). The standard concentrations fell within the upper and lower limits of sensitivity of the ATP bioluminescence assay. The ATP concentration in the cell supernatant was calculated relative to the standard curve. Luminescence was also measured for all concentrations of the nucleotides used. The luminescence determined for each concentration of nucleotide was used as a blank for that treatment and was subtracted from the corresponding cellular luminescence before the ATP concentration was calculated. Treatments were carried out in triplicate and the mean ATP concentration (in nM) per treatment determined.

### 2.3. EctoATPase Studies

Confluent urothelial cells in 24 well plates were washed (three times) in phosphate free media (containing mM: NaCl 120, KCl 5, CaCl_2_ 2, HEPES 20, and D-glucose 10) [12]. After washing cells were incubated with 100 *μ*M nucleotide. After 30 minutes the supernatant was collected and the phosphate concentration determined. In additional experiments confluent cells in 12 well plates were incubated in control or hypotonic (50% dilution in water) phosphate free media for 10 or 30 minutes and both ATP concentration and phosphate concentration determined.

Phosphate liberated from nucleotides was determined as previously described [21]. Equal volumes of cell supernatant and colour reagent (containing 1% ammonium molybdite, 0.3 M H_2_SO_4_, 4% FeSO_4_) were mixed. After 15 minutes absorbance at 750 nm was measured in a plate reader (BMG labtech Polarstar). Phosphate concentration in the cell supernatant was determined relative to a standard curve (KH_2_PO_4_, 10 to 150 nM). Treatments were carried out in triplicate and the mean phosphate concentration (in nM) per treatment determined.

### 2.4. Statistics

Results are nonnormally distributed and as such are expressed as median with interquartile range (IQR). Two different treatments were compared using a Mann-Whitney *t*-test. Concentration response relationships were examined using a sigmoidal concentration response curve. All statistics were performed using Graphpad Prims (version 6) (San Diego, CA).

### 2.5. Materials

All cell culture reagents were purchased from Invitrogen (Mount Waverley, Australia). Bioluminescence ATP Assay kit and nucleotides were from Sigma-Aldrich (Sydney, Australia). All other reagents were of high analytical grade.

## 3. Results

### 3.1. Effect of Nucleotides on Urothelial Cell ATP Release

Hypotonic Krebs was used as a stretch stimulus and was seen to induce an approximate threefold increase in ATP release (*P* = 0.0006). Incubation of RT4 cells with ADP (100 *μ*M for 10 minutes) induced ATP release to a level that was 40-fold higher compared to the hypotonic stimulus (Table 1, *P* < 0.0011). In the presence of AMP and adenosine, the level of ATP release was not significantly different to the control level (Table 1). The response to CTP and GTP was not significantly different to the ATP release induced by hypotonic media (Table 1).

**Figure 1:**
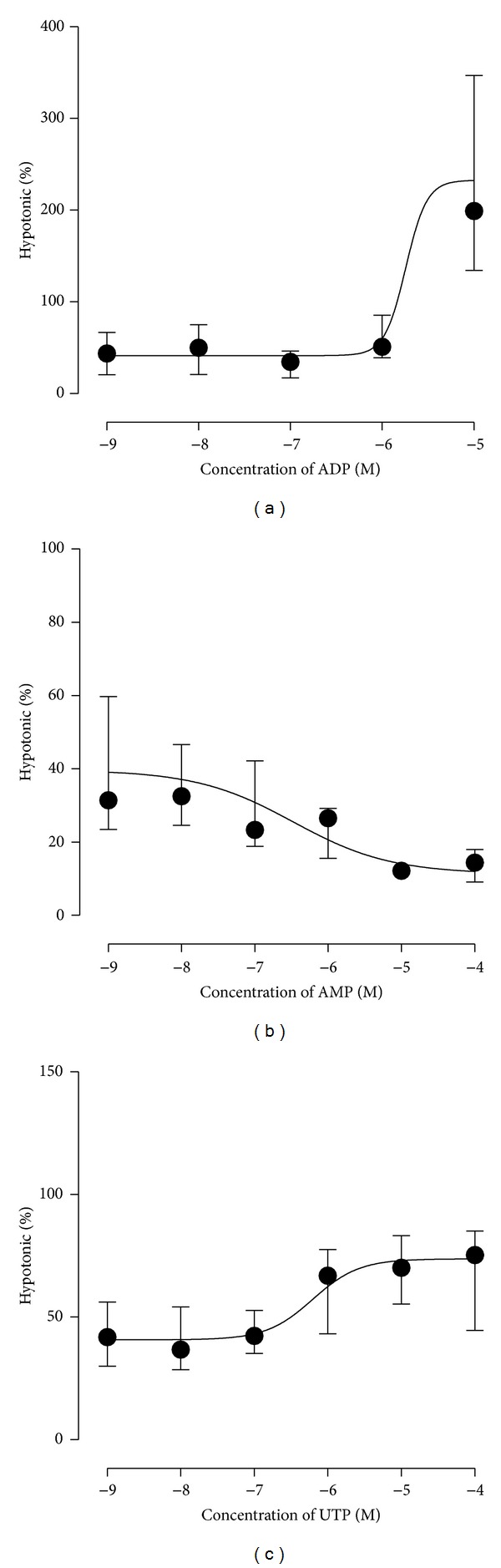
Concentration response relationships for nucleotides ADP (a), AMP (b), and UTP (c) on RT4 urothelial cell ATP release.

When the concentration response effect of ADP induced ATP release was determined it was seen to only occur at high concentrations of ADP (Figure 1(a)) with an EC50 of 1.8 *μ*M determined (*n* = 5). A concentration dependent inhibition of ATP release was seen with AMP (Figure 1(b)) with an EC50 of 0.33 *μ*M (*n* = 7). A concentration dependent increase in ATP release was observed with UTP, up to a maximum of approximately 70% of the hypotonic response (Figure 1(c)). An EC50 of 0.62 *μ*M was determined (*n* = 7).

**Figure 2:**
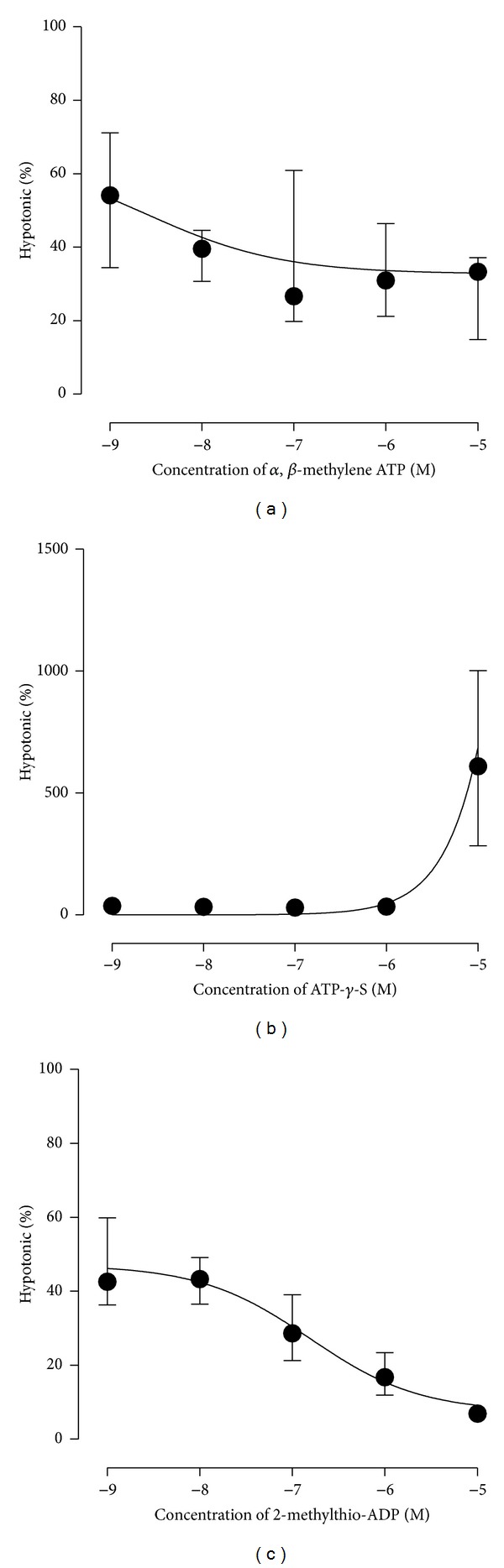
P2 receptor agonist stimulated ATP release. Incubation of RT4 urothelial cells with increasing concentration of *α*,*β*-methylene-ATP (a), ATP-*γ*-S (b), and 2-methylthio-ADP (c).

In RT4 cells, concentration response relationships were also determined for P2 receptor agonists and antagonists. The P2X agonist, *αβ*-methylene-ATP, and P2Y agonist, ATP-*γ*-S, had no effect on ATP release (Figures 2(a) and 2(b), *n* = 9 and 15, resp.). The more selective P2Y_1_ agonist, 2-methylthio-ADP, inhibited ATP release in a concentration dependent manner (Figure 2(c), EC50 0.16 *μ*M, *n* = 8).

P2 receptor antagonists were investigated for their ability to inhibit ADP-stimulated ATP release. PPADS (100 *μ*M) had no effect on ADP-stimulated ATP release (88.4 (65.2–113.4)% of ADP-stimulated release, *n* = 8, *P* = 0.4) while suramin (100 *μ*M) significantly inhibited ADP-stimulated ATP release (43.3 (27.6–41.7)% of ADP-stimulated ATP release, *P* = 0.002, *n* = 4; IC50 8.3 *μ*M, *n* = 8).

The results observed in RT4 urothelial cells were also confirmed in UROtsa cells (Table 2). Similar to the results observed in RT4 urothelial cells, treatment of UROtsa cells with hypotonic Krebs induced an increase in ATP release (*P* = 0.0087). Incubation of UROtsa cells with ADP (100 *μ*M for 10 minutes) induced an almost 50-fold increase in ATP release compared to that seen with the hypotonic stimulus (*P* < 0.0022). UTP (100 *μ*M) initiated ATP release similar to that seen with hypotonic stimulus while 2-methylthio-ADP (100 *μ*M) elicited an ATP release response similar to the control level.

### 3.2. EctoATPase Activity of Urothelial Cells

**Figure 3:**
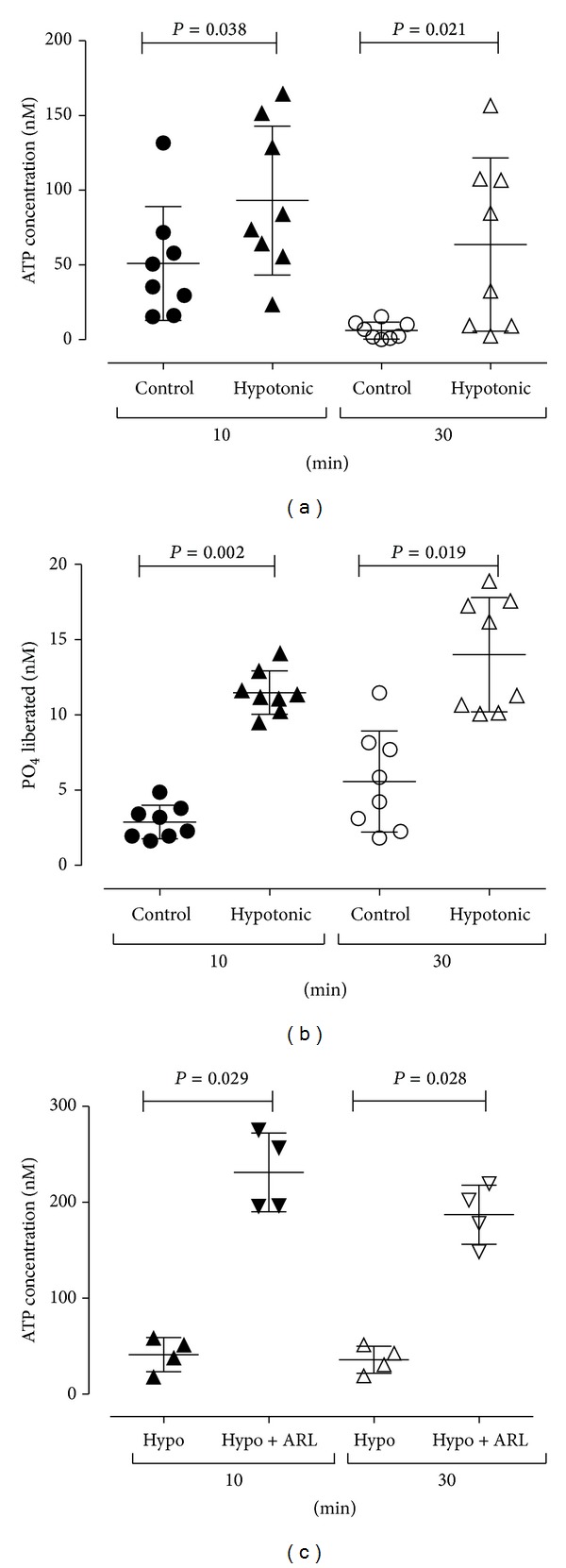
Phosphate liberation from endogenously released ATP. Incubation of RT4 urothelial cells in control or hypotonic phosphate free buffer for 10 or 30 minutes induced ATP release (a). Over the 10- or 30-minute incubation a small amount (approximately 10%) of this ATP was broken down by urothelial cells to liberate phosphate that could be detected (b). The presence of the ectoATPase inhibitor ARL67156 (100 *μ*M) was seen to increase the amount of ATP detected after both 10 and 30 minutes (c).

RT4 urothelial cells were shown to have the capacity to liberate phosphate from nucleotides (100 *μ*M), with the greatest amount of phosphate liberated from ADP and UTP (Table 3). In addition, a small amount of phosphate liberation was able to be demonstrated from endogenous nucleotides released during the experiment (Figure 3). When RT4 cells were incubated in either control or hypotonic phosphate free buffer for 10 or 30 minutes, significant ATP release was induced (Figure 3(a), *n* = 8). After a 10-minute incubation the level of release in the control was approximately 40 nM and in hypotonic phosphate free media was approximately 80 nM which was comparable to that seen using Krebs-Henseleit solution (Table 1). Over the 10- or 30-minute incubation some of this endogenous ATP was broken down by RT4 cells to liberate phosphate that could be detected (Figure 3(b), *n* = 8). Significantly more phosphate was detected in cells treated with hypotonic media for 10 and 30 minutes (*P* = 0.0002 and 0.0019, resp.). After a 10-minute incubation the level of phosphate liberated indicated that approximately 10% (7 to 15%) of the endogenously released ATP was broken down during the incubations in control and hypotonic media, respectively. In the presence of the ectoATPase inhibitor ARL67156 (100 *μ*M) the amount of ATP detected in cells treated with hypotonic phosphate free buffer increased 5-fold after both 10- and 30-minute incubations (Figure 3(c), *n* = 4).

## 4. Discussion

The demonstration of ATP release from the rabbit bladder urothelium in response to stretch by Ferguson and colleagues in 1997 [1] has been pivotal to our understanding of signalling within the bladder. However, our understanding of the factors that modulate ATP release and the autocrine signalling that occurs at the urothelial cell layer is limited. The current study has demonstrated that P2 receptor agonists are able to stimulate ATP release from urothelial cells. In fact the P2Y agonist ADP was shown in two urothelial cell lines, to stimulate a level of ATP release that is far in excess of that stimulated by hypotonic media. Similarly, UTP, another P2Y agonist [22], was also shown to stimulate ATP release, although to a lesser extent. In contrast, P2X agonists such as *α*,*β*-methylene-ATP were not seen to stimulate ATP release from cultured urothelial cells. These results are similar to the recently published findings by Sui and colleagues who reported that the P2Y agonist UTP stimulated ATP release in guinea pig and human bladder mucosal strips, while the P2X agonist *α*,*β*-methylene-ATP had no effect [22]. UTP stimulated ATP release has also been reported from primary cultures of rat bladder urothelial cells [17].

It has been shown that P2Y_1_ [5, 16], P2Y_2_ [5, 16, 17], P2Y_4_ [16, 17], and P2Y_11_ [5] receptor subtypes are present in bladder urothelial cells (see introduction). While P2X receptors respond preferentially to ATP, P2Y receptors are divided on the basis of their sensitivity to adenine nucleotides (P2Y_1_, P2Y_11_, P2Y_12_, and P2Y_13_) or uracil nucleotides (P2Y_2_, P2Y_4_, P2Y_6_, and P2Y_14_) [23]. Of the P2Y receptors that respond to adenine nucleotides, P2Y_1_, P2Y_12_, and P2Y_13_ respond to ADP while P2Y_11_ receptors respond to ATP [23]. In the current study, ATP release was stimulated by both ADP and UTP, although the release elicited by ADP was far greater than that elicited by UTP, suggesting the involvement of more than one P2Y receptor subtype. The EC50 value of ADP-stimulated ATP release suggests activity at P2Y_1_ or P2Y_11_ receptors. This is in agreement with previously reported EC50s at P2Y_1_ of 0.9–8 *μ*M [24–26]. The effects of UTP were closely associated with EC50 values at P2Y_2_ or P2Y_4_ receptors, agreeing with previously reported EC50s (0.14–0.8 *μ*M) at the P2Y_2_ receptor [24, 25] and P2Y_4_ receptor (2.5 *μ*M) [24, 25]. In contrast, the selective P2Y_1_ agonist, 2-methylthio-ADP [26], did not stimulate ATP release. The current study therefore suggests that more than one P2Y receptor subtype is likely to be involved in nucleotide stimulated ATP release. Unfortunately, the lack of subtype selective agonists for individual P2Y receptors makes it difficult to definitively identify the receptor subtypes involved in the observed responses.

It is possible that the apparent ATP release stimulated by high concentrations of ADP was due to the conversion of ADP to ATP by cell membrane-bound adenylate kinase. While this enzyme is predominately intracellular [27], it has been identified on the cell membrane of endothelial cells [28] and other cell types [27] although there have been no reports of cell membrane-bound adenylate cyclase in the urothelium. However, coincubation of ADP with the P2 receptor antagonist suramin inhibited ADP-stimulated ATP release, suggesting that it is indeed P2Y receptors that are involved in this response (reported IC50s 3–50 *μ*M) [15].

The source of ADP* in vivo* is unknown; however, nucleotides have recently been isolated from human urine samples [13]. In addition, urothelial cells express ectonucleotidase enzymes capable of degrading ATP and UTP to their respective nucleotides [29]. The basal and intermediate cells of the mouse bladder urothelium express NTPDase 3 [29]. Similarly, the human urothelial cell line RT4 used in the current study expresses NTPDase 3 and 5 [12] but not NTPDase 1 [12, 30]. NTPDase 1, 2, and 3 are known to face the extracellular environment and catalyse the breakdown of extracellular ATP [29]. It is thought that ectoATPase enzymes function to limit the exposure of P2 receptors to their ligands and to modulate the autocrine response to released ATP [29].

In the current study the activity of ectoATPase in urothelial RT4 cells was examined in three ways. Firstly, ectoATPase enzymes present on the RT4 urothelial cells were able to liberate phosphate from stretch-induced ATP release, indicating a capacity to generate mediators such as ADP from stretch-induced ATP release. Secondly, incubation of the ectoATPase inhibitor ARL67156 (100 *μ*M) [31] resulted in a higher level of stretch-induced ATP release being detected. At this concentration, ARL67156 has been shown to inhibit the activity of NTPDase3 (Ki 18 *μ*M) [32]. Finally, we demonstrated the capacity of ectoATPase enzymes associated with RT4 urothelial cells to liberate phosphate from exogenous nucleotides including ATP, ADP, AMP, CTP, GTP, and UTP. Similarly, Stella and associates showed that RT4 urothelial cells were able to liberate phosphate from exogenous nucleotides [12].

**Figure 4:**
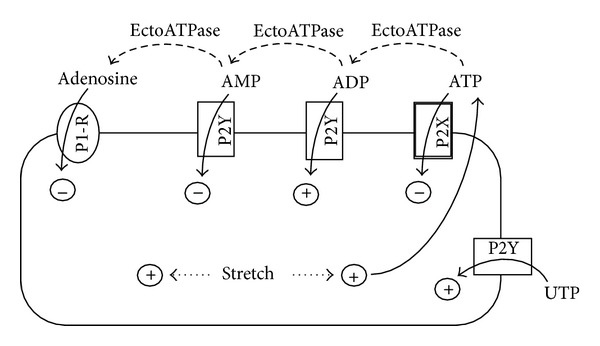
Hypothetical schematic representation of the complexity of the effect of nucleotides and stretch on urothelial cell ATP release.

The results obtained in the current study demonstrate that P2 receptor signalling in the urothelium is a complex interaction between ATP release and breakdown. To add to the complexity, it now seems likely that products formed from breakdown of nucleotides such as ATP also have affects at P2 receptors located on the urothelium. Our understanding of this complex interaction between ATP release and breakdown has been shown schematically in Figure 4. ADP has been previously postulated to stimulate urothelial ATP release [9] and to activate intracellular calcium transients [5, 33] that were greater than those activated by hypotonic solutions [33], a commonly used stimulus for ATP release. It was proposed that the ATP-dependent calcium transients were mediated by urothelial P2Y (rather than P2X) receptors [5, 33]; however, it is known that ATP has a low affinity at P2Y receptors [23, 25]. It is therefore possible that the calcium transients induced by endogenous ATP were in fact due to the generation of ADP by membrane-bound ectoATPases. This is supported by the findings of the current study which demonstrate that ADP is capable of stimulating ATP release. Adding to the complexity of P2 receptor signalling, further breakdown of ADP to AMP and adenosine was seen to inhibit ATP release. Inhibition of ATP release by adenosine has been previously reported in rabbit bladder mucosal strips [20]. These findings indicate that while the initial breakdown of ATP to ADP may exert positive feedback for ATP release which is short lived, further breakdown of ATP to AMP and adenosine may provide negative feedback for ATP release.

Activation of urothelial P2Y receptors has been proposed to be responsible for a number of physiological functions of the urothelium. Exogenous application of P2Y receptor agonists, including ADP and UTP, has been shown to increase spontaneous activity in rat bladder sheets [34]. Similar to the results seen in this study, it was unlikely that a single P2Y receptor subtype was responsible for the reported increase in spontaneous activity [34] with the involvement of P2Y_1_, P2Y_2_, P2Y_4_, and P2Y_6_ being hypothesised. In addition, activation of P2Y_2_ receptors leads to release of the inflammatory mediators interleukins 8 and 6 from uroepithelial cells [35], indicating a role for activation of P2Y receptors in inflammatory responses in the bladder. Interestingly, expression of the P2Y_2_ receptor decreased in a feline model of interstitial cystitis [16]. Intravesical instillation of a P2Y_6_ selective agonist induced bladder overactivity characterised by increased voiding frequency in a rat cystometry model in addition to increased ATP release into the voided fluid [19].

Alterations in urothelial ATP release have been identified in bladder dysfunction including interstitial cystitis [9], painful bladder syndrome [10, 11], and overactive bladder [3]. The results of this study demonstrate the complexity of P2 receptor signalling in the urothelium by elucidating a role for a number of P2Y receptor subtypes in initiating ATP release. Recent literature has shown the important role of these receptors in normal bladder physiology, indicating that these receptors may represent a potential future target for the treatments of bladder dysfunction.

## Figures and Tables

**Table 1:** ATP release in RT4 urothelial cells stimulated by 10-minute incubation with nucleotides (100 *µ*M).

	ATP release in RT4 cells
Control	20.28 (13.24–40.22) nM (*n* = 33)
Hypotonic	64.63 (43.75–92.95) nM (*n* = 33)
ADP	2641 (2006–3598) nM (*n* = 13)
AMP	8.56 (4.74–189.2) nM (*n* = 17)
Adenosine	5.58 (4.52–65.85) nM (*n* = 4)
CTP	51.12 (37.4–99.96) nM (*n* = 11)
GTP	55.33 (34.42–173.4) nM (*n* = 15)
UTP	60.57 (36.51–126.1) nM (*n* = 11)
*α*,*β*-Methylene-ATP	26.6 (22.5–77.5) nM (*n* = 9)
ATP-*γ*-S	2294 (429–2538) nM (*n* = 16)

**Table 2:** ATP release in UROtsa urothelial cells stimulated by 10-minute incubation with nucleotides (100 *µ*M).

	ATP release in UROtsa cells
Control	11.08 (4.3–30.2) nM (*n* = 6)
Hypotonic	68.3 (35.6–133.4) nM (*n* = 6)
ADP	3377 (752–6361) nM (*n* = 6)
UTP	61.2 (26.6–115.7) nM (*n* = 6)
2-Methylthio-ADP	17.7 (13.9–26.9) nM (*n* = 6)

**Table 3:** Phosphate liberation from 30-minute incubation of RT4 urothelial cells with 100 *µ*M nucleotides (*n* = 8).

	Phosphate liberated (nM)
ATP	1.3 (−0.9–3.8)
ADP	7.5 (1.9–12.1)
AMP	4.1 (3.3–7.7)
CTP	3.4 (1.2–4.8)
GTP	2.6 (0.3–6.2)
UTP	7.8 (6.5–9.9)

## References

[1] Ferguson DR, Kennedy I, Burton TJ (1997). ATP is released from rabbit urinary bladder epithelial cells by hydrostatic pressure changes—a possible sensory mechanism?. *The Journal of Physiology*.

[2] Cheng Y, Mansfield K, Sandow SL (2011). Porcine bladder urothelial, myofibroblast and detrusor muscle cells: characterisation and ATP release. *Frontiers in Pharmacology*.

[3] Cheng Y, Mansfield KJ, Allen W, Walsh CA, Burcher E, Moore KH (2010). Does adenosine triphosphate released Into voided urodynamic fluid contribute to urgency signaling in women with bladder dysfunction?. *The Journal of Urology*.

[4] Khakh BS, North RA (2006). P2X receptors as cell-surface ATP sensors in health and disease. *Nature*.

[5] Shabir S, Cross W, Kirkwood LA (2013). Functional expression of purinergic P2 receptors and transient receptor potential channels by human urothelium. *American Journal of Physiology: Renal Physiology*.

[6] Elneil S, Skepper JN, Kidd EJ, Williamson JG, Ferguson DR (2001). Distribution of P2X1 and P2X3 receptors in the rat and human urinary bladder. *Pharmacology*.

[7] Liu F, Takahashi N, Yamaguchi O (2009). Expression of P2X3 purinoceptors in suburothelial myofibroblasts of the normal human urinary bladder. *International Journal of Urology*.

[8] Vlaskovska M, Kasakov L, Rong W (2001). P2X3 knock-out mice reveal a major sensory role for urothelially released ATP. *The Journal of Neuroscience*.

[9] Sun Y, Chai TC (2006). Augmented extracellular ATP signaling in bladder urothelial cells from patients with interstitial cystitis. *American Journal of Physiology: Cell Physiology*.

[10] Kumar V, Chapple CR, Surprenant AM, Chess-Williams R (2007). Enhanced adenosine triphosphate release from the urothelium of patients with painful bladder syndrome: a possible pathophysiological explanation. *The Journal of Urology*.

[11] Cheng Y, Mansfield KJ, Allen W (2013). Correlation between cystometric volumes, ATP release, and pH in women with overactive bladder versus controls. *Neurourology and Urodynamics*.

[12] Stella J, Bavaresco L, Braganhol E (2010). Differential ectonucleotidase expression in human bladder cancer cell lines. *Urologic Oncology: Seminars and Original Investigations*.

[13] Contreras-Sanz A, Scott-Ward TS, Gilland HS (2012). Simultaneous quantification of 12 different nucleotides and nucleosides released from renal epithelium and in human urine samples using ion-pair reversed-phase HPLC. *Purinergic Signal*.

[14] Tatur S, Kreda S, Lazarowski E, Grygorczyk R (2008). Calcium-dependent release of adenosine and uridine nucleotides from A549 cells. *Purinergic Signalling*.

[15] von Kügelgen I (2006). Pharmacological profiles of cloned mammalian P2Y-receptor subtypes. *Pharmacology & Therapeutics*.

[16] Birder LA, Ruan HZ, Chopra B (2004). Alterations in P2X and P2Y purinergic receptor expression in urinary bladder from normal cats and cats with interstitial cystitis. *American Journal of Physiology: Renal Physiology*.

[17] Chopra B, Gever J, Barrick SR (2008). Expression and function of rat urothelial P2Y receptors. *American Journal of Physiology: Renal Physiology*.

[18] Sui G, Fry CH, Montgomery B (2013). Purinergic and muscarinic modulation of ATP release from the urothelium and its paracrine actions. *American Journal of Physiology: Renal Physiology*.

[19] Timóteo MA, Carneiro I, Silva I (2014). ATP released via pannexin-1 hemichannels mediates bladder overactivity triggered by urothelial P2Y6 receptors. *Biochemical Pharmacology*.

[20] Dunning-Davies BM, Fry CH, Mansour D (2013). The regulation of ATP release from the urothelium by adenosine and transepithelial potential. *BJU International*.

[21] Else PL, Windmill DJ, Markus V (1996). Molecular activity of sodium pumps in endotherms and ectotherms. *American Journal of Physiology: Regulatory Integrative and Comparative Physiology*.

[22] Sui G-P, Wu C, Fry CH (2006). Characterization of the purinergic receptor subtype on guinea-pig suburothelial myofibroblasts. *BJU International*.

[23] Abbracchio MP, Burnstock G, Boeynaems J-M (2006). International Union of Pharmacology LVIII: update on the P2Y G protein-coupled nucleotide receptors: from molecular mechanisms and pathophysiology to therapy. *Pharmacological Reviews*.

[24] Bours MJL, Swennen ELR, di Virgilio F, Cronstein BN, Dagnelie PC (2006). Adenosine 5′-triphosphate and adenosine as endogenous signaling molecules in immunity and inflammation. *Pharmacology & Therapeutics*.

[25] Jacobson KA, Balasubramanian R, Gao ZG (2012). G protein-coupled adenosine (P1) and P2Y receptors: ligand design and receptor interactions. *Purinergic Signal*.

[26] Waldo GL, Harden TK (2004). Agonist binding and Gq-stimulating activities of the purified human P2Y1 receptor. *Molecular Pharmacology*.

[27] Yegutkin GG (2008). Nucleotide- and nucleoside-converting ectoenzymes: important modulators of purinergic signalling cascade. *Biochimica et Biophysica Acta: Molecular Cell Research*.

[28] Quillen EE, Haslam GC, Samra HS (2006). Ectoadenylate kinase and plasma membrane ATP synthase activities of human vascular endothelial cells. *The Journal of Biological Chemistry*.

[29] Yu W, Robson SC, Hill WG (2011). Expression and distribution of ectonucleotidases in mouse urinary bladder. *PLoS ONE*.

[30] Mohlin C, Säve S, Nilsson M, Persson K (2009). Studies of the extracellular atp-adenosine pathway in human urinary tract epithelial cells. *Pharmacology*.

[31] Drakulich DA, Spellmon C, Hexum TD (2004). Effect of the ecto-ATPase inhibitor, ARL 67156, on the bovine chromaffin cell response to ATP. *European Journal of Pharmacology*.

[32] Lévesque SA, Lavoie ÉG, Lecka J, Bigonnesse F, Sévigny J (2007). Specificity of the ecto-ATPase inhibitor ARL 67156 on human and mouse ectonucleotidases. *British Journal of Pharmacology*.

[33] Wu C, Gui GP, Fry CH (2011). Intracellular Ca^2+^ regulation and electrophysiolgical properties of bladder urothelium subjected to stretch and exogenous agonists. *Cell Calcium*.

[34] Fry CH, Young JS, Jabr RI (2012). Modulation of spontaneous activity in the overactive bladder: the role of P2Y agonists. *American Journal of Physiology: Renal Physiology*.

[35] Kruse R, Säve S, Persson K (2012). Adenosine triphosphate induced P2Y2receptor activation induces proinflammatory cytokine release in uroepithelial cells. *The Journal of Urology*.

